# Supplementary material for: Mutation-specific CAR T cells as precision therapy for IGLV3-21R110 expressing high-risk chronic lymphocytic leukemia
Source: Nat Commun. 2024 Feb 2;15:993. doi: 10.1038/s41467-024-45378-w (PMC10837166; doi:10.1038/s41467-024-45378-w)
Supplement: Supplementary file 1 — Supplementary Information [file 41467_2024_45378_MOESM1_ESM.pdf]

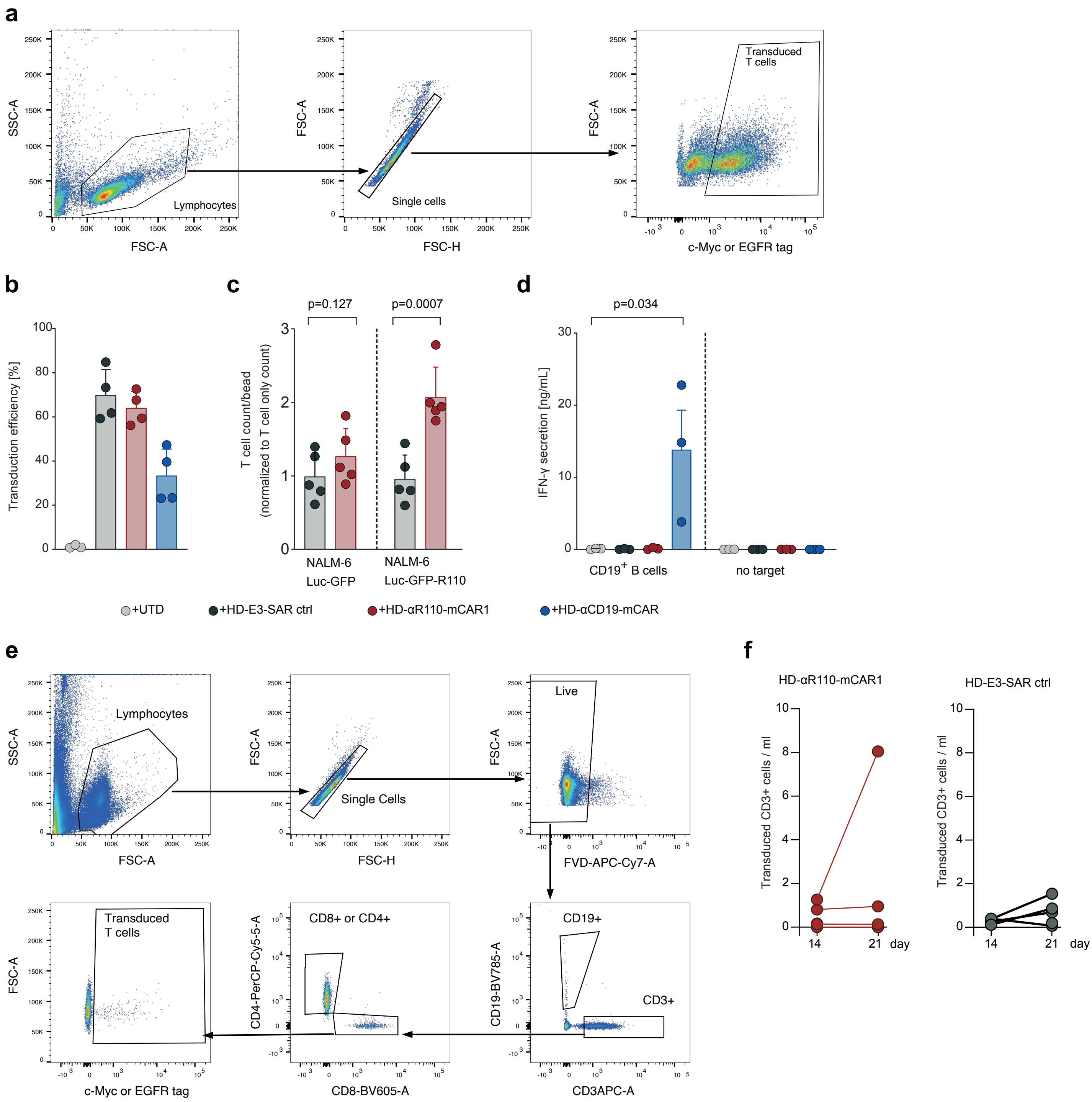

Supplementary Fig. 1 Generation and validation of a murine anti-IGLV3-21^{R110} CAR construct. a Gating strategy for the quantification of the transduction efficiency of the murine CAR T construct. T cells with surface expression of co-delivered c-Myc tag or EGFR were identified as CAR T cells. **b** Efficiency of the retroviral transduction of the murine CAR construct into human T cells. Bars represent mean of n=4 independent experiments. **c** Expansion of HD-αR110-mCAR1 T cells in co-culture with NALM-6 Luc-R110 in n=5 independent experiments. Following 48h of co-culture with NALM-6 Luc-R110, CD3^{+} T cell count per counting bead was assessed by flow cytometry. Counts were normalized to T cells grown as monoculture. Statistics: one-tailed unpaired t test. **d** Quantification of IFN-γ secretion in cell culture supernatants after 48h co-culture of CD19^{+} B cells with indicated CAR T cells. Statistics: one-tailed unpaired t test for =3 independent experiments. **e** Gating strategy for longitudinal tracking of i.v. injected HD-αR110-mCAR1 and E3-SAR ctrl CAR T cells (= transduced CD3+EGFR+ T cells) in xenograft models. **f** Tracking of HD-αR110-mCAR1 (n=5) and E3-SAR ctrl (n=5) CAR T cells in the blood of individual mice as quantified by flow cytometry.

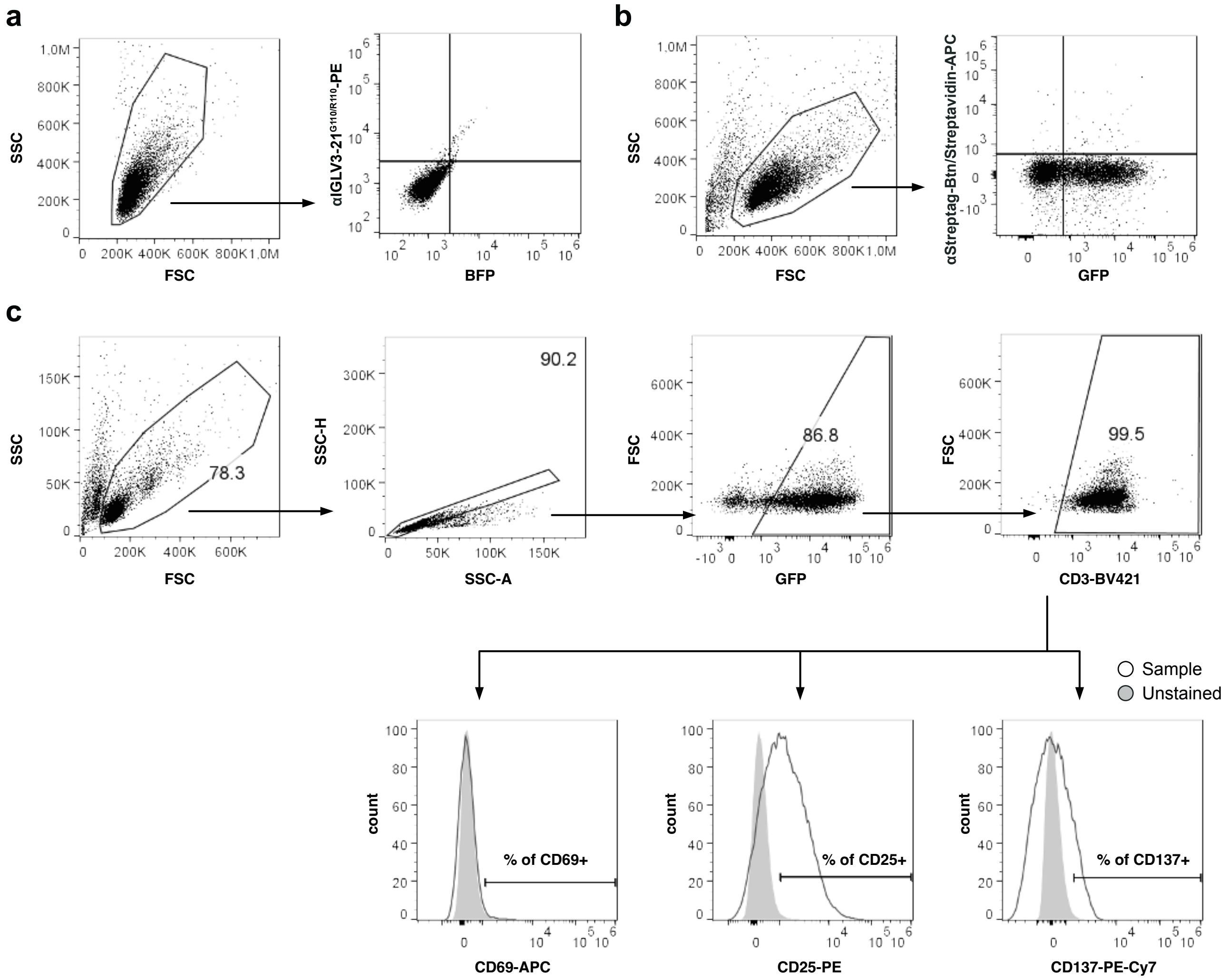

Supplementary Fig. 2 Specificity validation of the anti-IGLV3-21^{R110} CAR construct. a Gating strategy for validation of NALM-6-G110 and -R110 cell models. **b** Gating strategy for determining CAR T transduction efficiency. CAR T cells were identified by co-expression of an Strep-tag cloned between the scFv and the CD8 hinge in the CAR backbone and a co-delivered GFP. btn, biotin. **c** Gating strategy to determine surface expression of activation markers on CAR T cells after co-culture with target cells as presented in Fig. 2h-j.

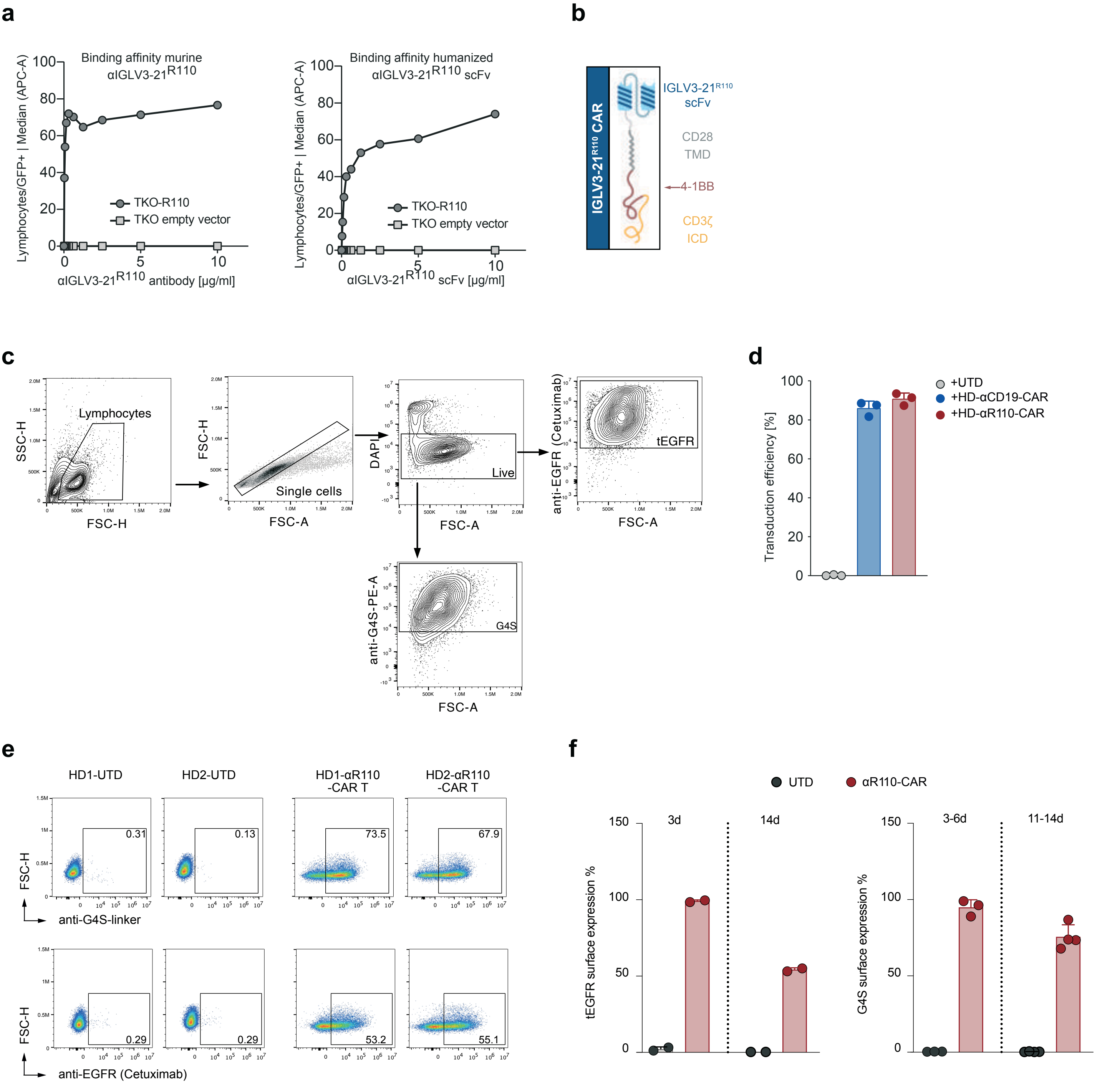

Supplementary Fig. 3 Humanization of the anti-IGLV3-21^{R110} CAR construct and cloning into a minimal-sized plasmid. a. Determination of median (n=3 independent samples) binding affinities of the murine anti-IGLV3-21^{R110} antibody or the corresponding humanized scFv construct. Serial dilutions were incubated with TKO cells expressing the IGLV3-21^{R110} antigen and binding intensity was quantified using flow cytometry. **b** Schematic representation of the humanized scFv fragment cloned into a 2nd generation CAR backbone with 4-1BB-CD3ζ costimulatory domains. Created with BioRender.com. **c** Gating strategy for the quantification of the transduction efficiency of the humanized CAR T constructs. T cells with surface expression of the G4S linker and the co-delivered truncated (t)EGFR were identified as CAR T cells. **d** Efficiency of the lentiviral transduction of the humanized CAR construct into primary human T cells. Bars represent the mean ± SD of n=3 independent experiments. **e-f** Efficiency of transposon-based CAR T cell generation from healhy (HD) T cells. Representative gating of G4S and EGFR surface expression **(e)** and quantification after 3-6 (n=2 for EGFR, n=3 for G4S) and 11-14 days (d) (n=2 for EGFR, n=5 for G4S) post-electroporation from indicated n independent experiments **(f).** Source data are provided as source data file.

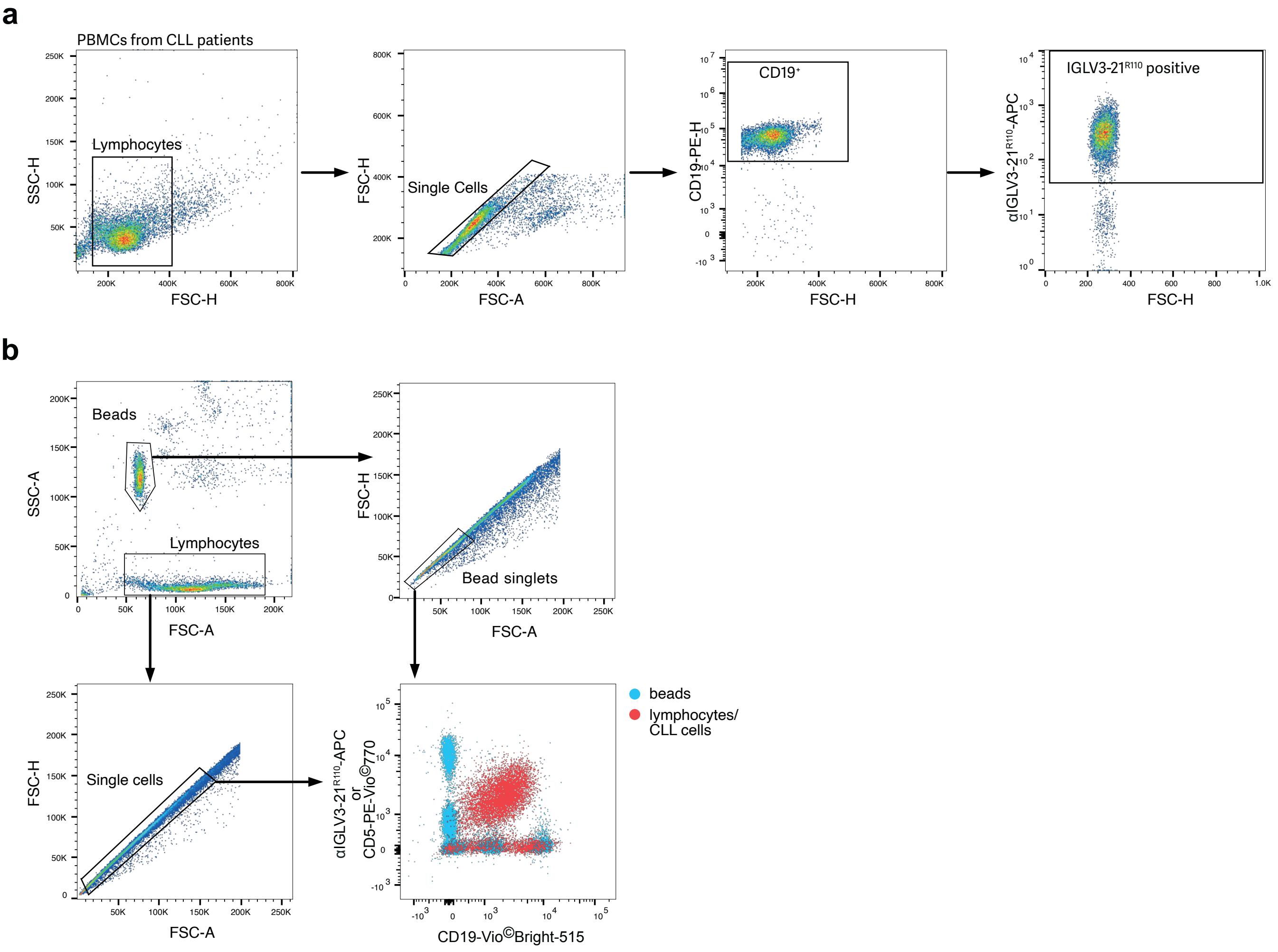

Supplementary Fig. 4 Screening of CLL patients for IGLV3-21^{R110} surface expression. a Gating strategy for detection of IGLV3-21^{R110} surface expression in CLL patients using the murine anti-IGLV3-21^{R110} antibody. **b** Gating strategy for bead-based detection of IGLV3-21^{R110} surface expression in CLL patients. Positive cases were identified as CD19+CD5+IGLV3-21+.

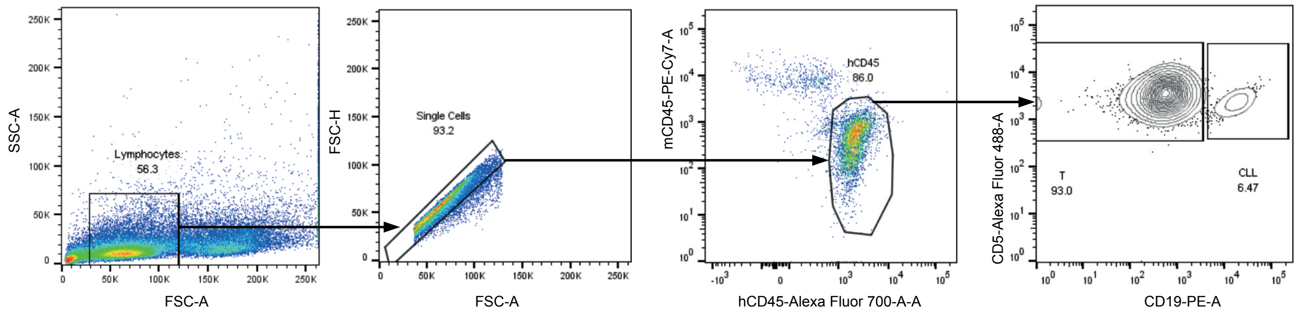

Supplementary Fig. 5 Gating strategies for the detection of human T cells and engrafted CLL cells in a patient-derived xenograft mouse model for CLL. Human lymphocytes were quanified after i.v. co-injection of T cells and CLL cells into NSG mice followed by CAR T treatment 10 days later. Single human lymphocytes were identified as mCD45-hCD45+. Within this population, CD5+CD19+ cells were identified as CLL cells, CD5-CD19+ cells as T cells.

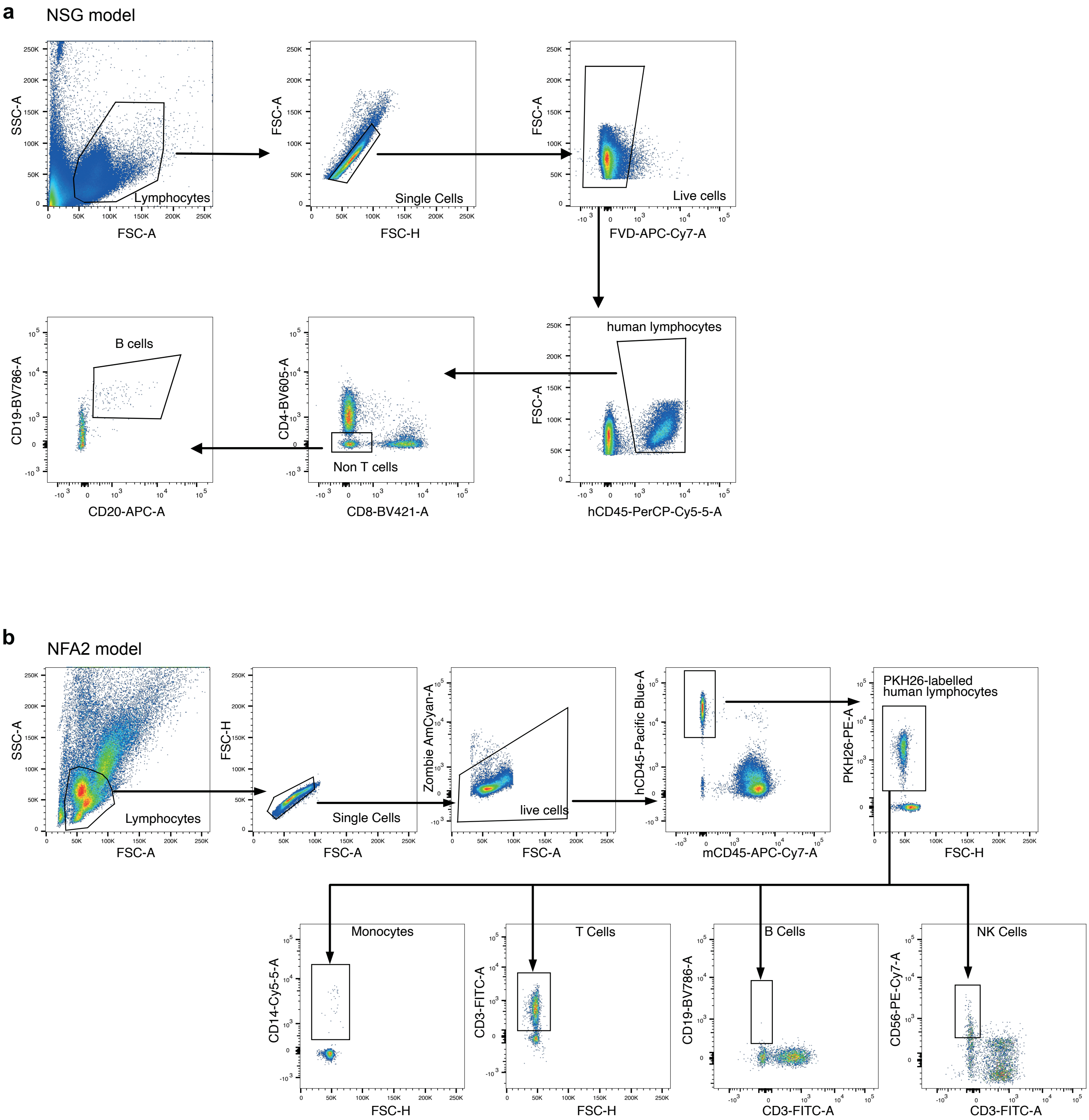

Supplementary Fig. 6 Gating strategies for the detection of human lymphocytes in humanized mouse models. a Gating strategy for quantification of B cells in the blood of NSG mice after i. v. injection of human PBMCs followed by CAR T treatment. Single live human B cells were identified as hCD45+CD4-CD8-CD19+CD20+. **b** Gating strategy for quantification of B cells in the peritoneal lavage after i. p. injection of PKH26-labelled human PBMCs and CAR T cells into NFA2 mice. Human PBMC subpopulations were identified as hCD45+PKH26+CD3-CD19+ (B cells), hCD45+PKH26+CD3-CD56+ (NK cells), hCD45+PKH26+CD3* (T cells) and hCD45+PKH26+CD14+ (Monocytes).

Supplementary table 1: ScFv sequences of αR110-CAR constructs.

Name	VH	VL	Reference
murine ScFv	QVQLQQSGPGLVQPSQSLSITCTVSGFSLTSYGIHWVRQSPGKGLEWLGVIWRGGGTDSNAAFMSRLSITKDNSKSQVFFKMNSLQADDTAIYYCARSRYDEEESMNYWGQGTSVTVSS	QIVLTQSPASLSASVGETVTITCRASGNIHSYLAWYQQKQGKSPQLLVYNAKTLADGVPSRFSGSGSGTQYSLKINSLQPEDFGSYYCQHFWNTPPTFGAGTKLELK	Publication Number: WO/2019/008129
humanized ScFv	QVQLQESGPGLVKPSETLSLTCTVSGFSLTSYGIHWIRQSPGRGLEWIGVIWRGGGTDSNAAFMSRITISRDTSKTQVSLKLGSVTAADTAIYYCARSRYDEEESMNYWGQGTSVTVSS	EIVLTQSPSSLSASVGDSVTITCRASGNIHSYLAWYQQKPGKAPKLLIYNAKTLADGVPSRFSGSGSGTQYTLTISSLQPEDFATYYCQHFWNTPPTFGAGTKLELK	Application Number: EP22156205.1 / EP22186810.2
