# Supplementary material for: Mutation-specific CAR T cells as precision therapy for IGLV3-21R110 expressing high-risk chronic lymphocytic leukemia
Source: Nat Commun. 2024 Feb 2;15:993. doi: 10.1038/s41467-024-45378-w (PMC10837166; doi:10.1038/s41467-024-45378-w)
Supplement: Supplementary file 3 — Reporting Summary [file 41467_2024_45378_MOESM3_ESM.pdf]

nature portfolio

Corresponding author(s): Mascha Binder
Sebastian Kobold

Last updated by author(s): Jan 15, 2024

Reporting Summary

Nature Portfolio wishes to improve the reproducibility of the work that we publish. This form provides structure for consistency and transparency in reporting. For further information on Nature Portfolio policies, see our Editorial Policies and the Editorial Policy Checklist.

Statistics

For all statistical analyses, confirm that the following items are present in the figure legend, table legend, main text, or Methods section.

n/a	Confirmed	
☐	☒	The exact sample size (*n*) for each experimental group/condition, given as a discrete number and unit of measurement
☐	☒	A statement on whether measurements were taken from distinct samples or whether the same sample was measured repeatedly
☐	☒	The statistical test(s) used AND whether they are one- or two-sided<br>*Only common tests should be described solely by name; describe more complex techniques in the Methods section.*
☒	☐	A description of all covariates tested
☐	☒	A description of any assumptions or corrections, such as tests of normality and adjustment for multiple comparisons
☐	☒	A full description of the statistical parameters including central tendency (e.g. means) or other basic estimates (e.g. regression coefficient) AND variation (e.g. standard deviation) or associated estimates of uncertainty (e.g. confidence intervals)
☐	☒	For null hypothesis testing, the test statistic (e.g. *F*, *t*, *r*) with confidence intervals, effect sizes, degrees of freedom and *P* value noted<br>*Give P values as exact values whenever suitable.*
☒	☐	For Bayesian analysis, information on the choice of priors and Markov chain Monte Carlo settings
☒	☐	For hierarchical and complex designs, identification of the appropriate level for tests and full reporting of outcomes
☒	☐	Estimates of effect sizes (e.g. Cohen's *d*, Pearson's *r*), indicating how they were calculated

Our web collection on statistics for biologists contains articles on many of the points above.

Software and code

Policy information about availability of computer code

Data collection	No software was used for data collection
Data analysis	The following algorithms/databases were used for analysis of sequencing data:<br>The MiXCR framework v3.0.12 and the IMGT v3 IGH library<br>The following software was used for data analysis:<br>GraphPd Prism software (v8.3.0)

For manuscripts utilizing custom algorithms or software that are central to the research but not yet described in published literature, software must be made available to editors and reviewers. We strongly encourage code deposition in a community repository (e.g. GitHub). See the Nature Portfolio guidelines for submitting code & software for further information.

Data

Policy information about availability of data

All manuscripts must include a data availability statement. This statement should provide the following information, where applicable:

- Accession codes, unique identifiers, or web links for publicly available datasets
- A description of any restrictions on data availability
- For clinical datasets or third party data, please ensure that the statement adheres to our policy

Sequences of humanized scFVs are subject to ongoing patent application (Application Number: EP22156205.1 / EP22186810.2). NGS data is deposited at the

European Nucleotide Archive (ENA) under the accession number PRJEB65274. All other data supporing the findings of this study are provided with this paper. Raw data is available from the corresponding authors upon request.

Research involving human participants, their data, or biological material

Policy information about studies with human participants or human data. See also policy information about sex, gender (identity/presentation), and sexual orientation and race, ethnicity and racism.

Reporting on sex and gender	Sex and gender was not relevant for the finding and interpretation of this study.
Reporting on race, ethnicity, or other socially relevant groupings	There are no socially constructed or socially relevant categorization variable(s) used in this manuscript.
Population characteristics	The CLL patient cohort sampled to profile for IGLV3-21R110 cases had a median age of 67 years (range 43-86) and consisted to 69% of male and 31% female participants. All patients provided written informed consent for study participation.
Recruitment	The patients with confirmed CLL diagnosis were recruited at the Universities of Hamburg–Eppendorf, Freiburg and Halle-Wittenberg, Germany.
Ethics oversight	Study was approved by the ethics committees of the Universities of Hamburg–Eppendorf, Freiburg and the Martin-Luther-University Halle-Wittenberg, Halle (Saale), Germany. The study is conducted according to the ethical principles of the Declaration of Helsiniki, Good Clinical Practice and applicable regulatory requirements.

Note that full information on the approval of the study protocol must also be provided in the manuscript.

Field-specific reporting

Please select the one below that is the best fit for your research. If you are not sure, read the appropriate sections before making your selection.

- [x] Life sciences
- [ ] Behavioural & social sciences
- [ ] Ecological, evolutionary & environmental sciences

For a reference copy of the document with all sections, see nature.com/documents/nr-reporting-summary-flat.pdf

Life sciences study design

All studies must disclose on these points even when the disclosure is negative.

Sample size	No statistical methods were used to pre-determine sample sizes. The size of the CLL cohort for IGLV3-21-R110 screening was chosen based on earlier reports of 10-15% positive patients in unselected populations.
Data exclusions	In the humanized NFA2 mouse model, mice in which either PKH26-labelled PBMC or CAR T cells could not be detected in peritoneal lavage samples by flow cytometry were excluded from the dataset to control for unsuccessful i.p. injections. No other data were excluded from the analysis.
Replication	All cell-based experiments were performed in biological triplicates.<br>Experiments with murine models were separated in cohorts with at least 6 independent mice per condition.<br>To ensure the general functionality of the reported scFv fragment, different CAR backbones and model systems were used. In addition, experiments were performed independently at different laboratory sites: The NALM-6/OCI-Ly1 xenograft experiments as well as the humanized NSG experiments were performed in the Kobold lab, the NALM-6-G110 (wt) and -R110 assays in the Minguet lab, the CLL xenograft experiments at the Chiorazzi lab, the humanized NFA2 mouse model in the Chijioke lab and all other experiments at the Binder lab. All attempts at replication were successful.
Randomization	For the NALM-6 xenograft model, NSG mice were randomized into treatment groups according to tumor burden.<br>For the CLL xenograft model, mice were randomly split into two groups: 6 mice were intraperitoneal injected with 7 million CAR T cells or untransduced T cells (UTD) generated from the same patient. Randomization of cell-based in vitro experiments is not applicable.
Blinding	Experiments with mice were performed by a scientist blinded to treatment allocation and with adequate controls. Screening of CLL patients was performed batch-wise blinded together with samples from healthy control individuals. Blinding of cell-based in vitro experiments is not applicable.

Reporting for specific materials, systems and methods

We require information from authors about some types of materials, experimental systems and methods used in many studies. Here, indicate whether each material, system or method listed is relevant to your study. If you are not sure if a list item applies to your research, read the appropriate section before selecting a response.

Materials & experimental systems

n/a	Involved in the study
	[x] Antibodies
	[x] Eukaryotic cell lines
[x]	Palaeontology and archaeology
	[x] Animals and other organisms
[x]	Clinical data
[x]	Dual use research of concern
[x]	Plants

Methods

n/a	Involved in the study
[x]	ChIP-seq
	[x] Flow cytometry
[x]	MRI-based neuroimaging

Antibodies

Antibodies used

anti-CD3-APC (clone OKT3, BioLegend, #317318),
anti-CD4-PerCP/Cy5.5 (clone OKT4, BioLegend, #317428),
anti-CD8a-BV605 (clone RPA-T8, BioLegend, #301040),
anti-CD19-BV785 (clone 6D5, BioLegend, #115543),
anti-EGFR-AlexaFluor488 (clone AY13, BioLegend, #352901),
anti-c-Myc-FITC (clone SH1-26E7.1.3, Miltenyi Biotech, Bergisch-Gladbach, Germany, #130-116-485),
anti-CD20-APC (clone 2H7, BioLegend, #302309),
anti-CD4-BV605 (clone OKT4, BioLegend, #317438),
anti-CD8 BV421 (clone SK1, BioLegend, #344748),
anti-CD45 PerCP-Cy5.5 (clone 2D1, BioLegend, #368504),
anti-Biotin Strep-Tag (NWSHPQFEK) (clone 5A9F9, Genscript, #A01736),
anti-Streptavidin-APC (BioLegend #405207),
anti-hCD25-PE (clone BC96, BioLegend, #302606),
anti-hCD137-PE-Cy7 (clone 4B4-1, Invitrogen, #25-1379-42),
anti-hCD69-APC (clone CH/4, Invitrogen, #MHCD6905),
anti-hCD3-BV421 (clone UCHT1, BioLegend, #300434),
anti-human (h)CD45-Pacific Blue (2D1, Biolegend, San Diego, USA, #368540),
anti-mouse (m)CD45-APC (30-F11, Biolegend, #103114),
anti-human CD19-BV785 (HIB19, Biolegend, #302240),
anti-human CD3-FITC (UCHT1, Biolegend, #300406),
anti-human CD14-Cy5.5 (TuK4, Invitrogen, Carlsbad, USA, #MHCD1418),
anti-human CD56-PE-Cy7 (NCAM16.2, BD Franklin Lakes, USA, #335791),
anti-mCD45-PE-Cy7 (clone 30-F11, Biolegend, #103114),
anti-hCD45-AlexaFluor700 (clone HI30, Biolegend, #304024),
anti-CD19-PE (clone HIB19, Biolegend, #982402),
anti-CD5-AlexaFluor488 (clone UCHT2, Biolegend, #300632),
anti-IGLV3-21-R110-APC/PE (AVA Liefescience),
anti-EGFR (Cetuximab) (MerckKGaA),
anti-G4S-Linker (E7O2V, Cell Signaling Technologies, Danvers, USA, #63670),
anti-human IgG-FITC (Sigma-Aldrich #F4512),
anti-rabbit-IgG H&L-FITC (Abcam, Cambridge, UK, #ab6717),
anti-human-IgG1-APC (Clone IS11-12E4.23.20, Miltenyi, Bergisch Gladbach, Germany, #130-119-944).

The IGLV3-21-R110/G110-specific antibody (#02P0150) was obtained from AVA Lifescience GmbH, Denzlingen, Germany. The IGLV3-21-R110-specific antibody as well as an anti-CD5 and an anti-CD19 antibodies were part of the applied ApLife FastScreen CLL flow cytometry Kit (#AVA-FS001) from AVA Lifescience GmbH, Denzlingen, Germany.
Labeling:
anti-IGLV3-21-R110/G110-PE
anti-IGLV3-21-R110-APC/PE
anti-CD19-Vio©Bright515
anti-CD5-Vio©770

Validation

Specificity of the IGLV3-21R110-specific antibody was validated by sequencing of all tested CLL cases and by binding assays using cell lines engineered to express the epitope.
All other applied antibodies are commercially available with data sheet and validation information available from the respective supplier.

Eukaryotic cell lines

Policy information about cell lines and Sex and Gender in Research

Cell line source(s)

OCI-Ly1 (ACC 722) and NALM-6 (ACC 128) were purchased from the DSMZ (German Collection of Microorganisms and Cell Cultures GmbH)

Authentication	STR analysis
Mycoplasma contamination	Cell lines were regularly screened for mycoplasma contimination every 2-3 months. We test using the PCR-based MycoSPY Detection Kit from Biontex, Munich, Germany. No contaminations were detected.
Commonly misidentified lines (See ICLAC register)	No commonly misidentified cell lines (according to ICLAC register) were used.

Animals and other research organisms

Policy information about studies involving animals; ARRIVE guidelines recommended for reporting animal research, and Sex and Gender in Research

Laboratory animals	For NALM-6 and OCI-Ly1 xenografts and engraftment of human PBMCs: female NSG (NOD.Cg-Prkdcscid Il2rgtm1WjI/SzJ) mice, 2-3 months old (n = 85). For CLL xenograft: NOD/SCID/IL2rγnull (NSG) mice (n = 12), 8 weeks old.<br>For engraftment of human PBMCs: NFA2 mice (NOD.Cg-Rag1tm1Mom Flt3tm1Irl Mcph1Tg(HLA-A2.1)1Enge Il2rgtm1Wjl/J), 3-5 months old (n = 18).
Wild animals	No wild animals were used in this study.
Reporting on sex	Sex was not considered in the study design. Since most of the experiments with the exception of the NFA2 model were conducted in female mice only, no sex-based analysis was performed.
Field-collected samples	No field-collected samples were used in this study.
Ethics oversight	Animal studies were performed in accordance with experimental protocols approved by the Institutional Animal Care and Use Committee (IACUC) of the Feinstein Institute for Medical Research, the Gonvernment of Oberbayern and the veterinary office of the canton of Zurich, Switzerland (protocol ZH049/20).

Note that full information on the approval of the study protocol must also be provided in the manuscript.

Plants

Seed stocks	*Report on the source of all seed stocks or other plant material used. If applicable, state the seed stock centre and catalogue number. If plant specimens were collected from the field, describe the collection location, date and sampling procedures.*
Novel plant genotypes	*Describe the methods by which all novel plant genotypes were produced. This includes those generated by transgenic approaches, gene editing, chemical/radiation-based mutagenesis and hybridization. For transgenic lines, describe the transformation method, the number of independent lines analyzed and the generation upon which experiments were performed. For gene-edited lines, describe the editor used, the endogenous sequence targeted for editing, the targeting guide RNA sequence (if applicable) and how the editor was applied.*
Authentication	*Describe any authentication procedures for each seed stock used or novel genotype generated. Describe any experiments used to assess the effect of a mutation and, where applicable, how potential secondary effects (e.g. second site T-DNA insertions, mosiacism, off-target gene editing) were examined.*

Flow Cytometry

Plots

Confirm that:

- [x] The axis labels state the marker and fluorochrome used (e.g. CD4-FITC).
- [x] The axis scales are clearly visible. Include numbers along axes only for bottom left plot of group (a 'group' is an analysis of identical markers).
- [ ] All plots are contour plots with outliers or pseudocolor plots.
- [ ] A numerical value for number of cells or percentage (with statistics) is provided.

Methodology

Sample preparation	Peripheral mononuclear cells (PBMCs) were isolated by Ficoll gradient centrifugation, resuspended in FCS + 10% DMSO and stored in liquid nitrogen until further use. For measurement, samples were thawed, washed three times with PBS pH 7.2 and counted. 3 million cells were resuspended and stained according to the instructions of the ApLifeTM FastScreenCLL Assay (AVA Lifescience GmbH, Denzlingen, Germany). For staining of cell surface markers, cells were washed once with PBS supplemented with 2% FBS before antibody incubation for 15-30 minutes at 4°C.
Instrument	BD FACSCelesta, BD LSRFortessa, MACSQuant Analyzer 10 flow cytometer, Attune NxT Acoustic Focusing Cytometer (Invitrogen)
Software	FlowJo 10.8

Cell population abundance	No post-sort fraction was collected.
Gating strategy	For CLL screening, gating was performed according to the instructions of the ApLifeTM FastScreenCLL Assay (AVA Lifescience GmbH, Denzlingen, Germany). Cases with more than 20% of cells within the APC gate were considered positive for IGLV3-21R110. Gating strategies for all other flow cytometry assays are provided as Supplementary Figures.

☐ Tick this box to confirm that a figure exemplifying the gating strategy is provided in the Supplementary Information.
